# On the Uncertainty Properties of the Conditional Distribution of the Past Life Time

**DOI:** 10.3390/e25060895

**Published:** 2023-06-02

**Authors:** Mohamed Kayid, Mansour Shrahili

**Affiliations:** Department of Statistics and Operations Research, College of Science, King Saud University, P.O. Box 2455, Riyadh 11451, Saudi Arabia; msharahili@ksu.edu.sa

**Keywords:** coherent system, past entropy, Shannon differential entropy, system signature

## Abstract

For a given system observed at time *t*, the past entropy serves as an uncertainty measure about the past life-time of the distribution. We consider a coherent system in which there are *n* components that have all failed at time *t*. To assess the predictability of the life-time of such a system, we use the signature vector to determine the entropy of its past life-time. We explore various analytical results, including expressions, bounds, and order properties, for this measure. Our results provide valuable insight into the predictability of the coherent system’s life-time, which may be useful in a number of practical applications.

## 1. Introduction

The process of quantifying and managing uncertainty over the random life-time of a system is a major task for engineers. As uncertainty increases, the reliability of a system will also decrease, so systems that have a longer life-time while benefiting from a lower level of uncertainty are preferable (see, e.g., Ebrahimi and Pellery [1]). The concept of uncertainty has far-reaching applications, as highlighted in Shannon’s seminal work on information theory [2]. Information theory has provided valuable tools for evaluating and managing uncertainty in engineering systems. Let *X* be the lifespan of a system or other living organism with an absolutely continuous cumulative distribution function (cdf) F(x) and a probability density function (pdf) f(x). Shannon’s differential entropy is a well-known measure and is given as follows:

(1) $$H\left(X\right)=-\int_{0}^{\infty }f\left(x\right)\log f\left(x\right)dx,$$

where “log” stands for the natural logarithm. If *X* represents the life-time a new system has, then H(X) calculates the uncertainty for the life-time of the system. In certain scenarios, operators may only partially know the age that a system currently has. For example, an operator may know that a system was in service at specified time *t*, and he/she is quantifying the uncertainty for the remaining life-time of the system after age *t*, commonly referred to as the remaining life-time or residual life-time after *t*. According to Ebrahimi [3], the residual entropy of *X* is considered to be the entropy of $X^{t}=\left[X-t|X>t\right]$. Formally, for all t>0, the residual life-time entropy for *X* is measured as

(2) $$H\left(X^{t}\right)=-\int_{t}^{\infty }\frac{f(x)}{1-F(t)}\log\frac{f(x)}{1-F(t)}dx,$$

If we already know that an object has survived to time t, then $H(X^{t})$ quantifies the uncertainty contained in the distribution of remaining life-times. Di Crescenzo and Longobardi [4] have proposed a notion of past entropy over the interval (0,t) using an analogy with the definition of entropy over time given in Equation (2). The introduction of the past entropy is motivated by the observation that in realistic scenarios, uncertainty needs not be limited to the future, but can also affect the past. The authors pointed out the importance of the past entropy and its relation to the residual entropy. Thus, if *X* is a random life-time and recalls that the pdf of $X_{t}=\left[X|X<t\right]$ is $f_{t}\left(x\right)=f\left(x\right)/F\left(t\right),\;0<x<t,$ then the differential entropy of [X|X<t] is called the past entropy at time *t* of X, and it is denoted by

(3) $$\begin{array}{ccc} \bar{H}\left(X_{t}\right) & = & -\int_{0}^{t}\frac{f(x)}{F(t)}\log\frac{f(x)}{F(t)}dx\\ & = & 1-\frac{1}{F(t)}\int_{0}^{t}f\left(x\right)\log\tau \left(x\right)dx, \end{array}$$

where τ(x)=f(x)/F(x) is known as the reversed hazard rate function.

Various aspects and statistical perspectives on past entropy have been treated in the literature, as can be seen in Di Crescenzo and Longobardi [4], Nair and Sunoj [5], Loperfido [6], and Shangari and Chen [7], as well as in the references used in these papers. In this case, Gupta et al. [8] obtained some results concerning the residual entropy and past entropy for order statistics, as well as presented several relevant stochastic ordering properties. In this context, they provided some characterization properties; see also [9]. Recently, Toomaj et al. [10] applied the residual entropy to a coherent system and obtained several related properties. Kayid and Alshehri [11] have recently studied the uncertainty in coherent structures using Tsallis entropy. In addition, Mesfioui et al. [12] also studied the phenomenon of uncertainty in the life-time of a coherent system using the Rényi entropy. In this research, we consider a coherent structure where all of the components have failed at time t. The system signature approach is utilized to compute the differential entropy of the past life-time.

The contents of this paper are organized as follows: In Section 2, we present a formula for the Shannon differential entropy of a coherent system when all components are inactive at time t. The method of system signature is applicable when the random life-times of the components are independent and identically distributed (i.i.d.). In Section 3, some valuable bounds are pointed out and outlined. In Section 4, the Jensen–Shannon disparity of the coherent framework is considered. Some concluding remarks are outlined in Section 5.

## 2. The Past Life-Time Uncertainty in Coherent Systems

Here, in order to define the past-life entropy for coherent structures, we apply the signature vector of the underlying structure. We assume that all of the components in the system have become inactive at time t. The coherent system is defined as a system that satisfies the requirements of having no unnecessary components and has a monotonic structure function. The vector $p=(p_{1},\ldots ,p_{n})$, in which the *i*th component is given by $p_{i}=P\left(T=X_{i:n}\right),\;i=1,2,\ldots ,n,$ is known as the signature vector (see [13]). We contemplate a coherent structure with components that have i.i.d. random life-times $X_{1},\ldots ,X_{n}$ and a specified signature $p=(p_{1},\ldots ,p_{n})$. If $T_{t}=\left[t-T|X_{n:n}\leq t\right]$ stands for the past life-time of the system, provided that at time *t* the components have all become inactive, then from the results of Khaledi and Kochar [14], the survival function of $T_{t}$ can be obtained as

(4) $$P\left(T_{t}>x\right)=\sum_{i=1}^{n}p_{i}P\left(t-X_{i:n}>x|X_{n:n}\leq t\right),$$

where

P(t−Xi:n>x|Xn:n≤t)=∑k=innkF(t−x)F(t)k1−F(t−x)F(t)n−k,0<x<t,

denotes the survival function of the past life-time of an *i*-out-of-*n* system as long as all of the components have failed at time *t*. It follows from (4) that

(5) $$f_{T_{t}}\left(x\right)=\sum_{i=1}^{n}p_{i}f_{T_{t}^{i}}\left(x\right),$$

where

(6) $$f_{T_{t}^{i}}\left(x\right)=\frac{\Gamma (n+1)}{\Gamma (i)\Gamma (n-i+1)}{\left(\frac{F(t-x)}{F(t)}\right)}^{i-1}{\left(1-\frac{F(t-x)}{F(t)}\right)}^{n-i}\frac{f(t-x)}{F(t)},\;0<x<t,$$

such that Γ(·) is the full gamma function and $T_{t}^{i}=\left[t-X_{i:n}|X_{n:n}\leq t\right],\;i=1,2,\cdots ,n,$ is the time elapsed since the failure of the component with life-time $X_{i:n}$ in the system, assuming that the system failed at or before time t. Remark that $T_{t}^{i}$ denotes the *i*-th order statistic among *n* i.i.d. components with cdf $\frac{F(t-x)}{F(t)},\;0<x<t.$ Now, we give a statement about the entropy of $T_{t}.$ To this aim, let us set $F_{t}\left(x\right)=\frac{F(x)}{F(t)},\;0<x<t.$ The probability integral transformation given by $V=F_{t}\left(T_{t}\right)$ plays a vital role in our study and it is obvious that $U_{i:n}=F_{t}\left(T_{t}^{i}\right)$ follows the beta distribution with parameters *i* and n−i+1 with pdf

(7) $$g_{i}\left(u\right)=\frac{\Gamma (n+1)}{\Gamma (i)\Gamma (n-i+1)}u^{i-1}{\left(1-u\right)}^{n-i},\;0<u<1,$$

for all i=1,⋯,n. In the forthcoming theorem, we shall give a formula for the past entropy of $T_{t}$ using (6).

**Theorem 1.**  *The past entropy of $T_{t}$ can be expressed as follows:*

(8) $$\begin{array}{ccc} \bar{H}\left(T_{t}\right) & = & H\left(V\right)-E[\log f_{t}\left(T_{t}\right)] \end{array}$$

(9) $$\begin{array}{ccc} & = & H\left(V\right)-\sum_{i=1}^{n}p_{i}E\left[\log f_{t}\left(F_{t}^{-1}\left(U_{i:n}\right)\right)\right], \end{array}$$

*V is the life-time of the coherent system which has pdf $g_{V}\left(v\right)=\sum_{i=1}^{n}p_{i}g_{i}\left(v\right)$ and $F_{t}^{-1}\left(u\right)=\inf\left\{x;F_{t}\left(x\right)\geq u\right\}$ is the quantile function of $F_{t}\left(x\right)=F\left(x\right)/F\left(t\right),\;0<x\leq t.$*

**Proof.**  By (1) and (6), and by substituting z=t−x, we have

(10) $$\begin{array}{ccc} \bar{H}\left(T_{t}\right) & = & -\int_{0}^{t}f_{T_{t}}\left(x\right)\log f_{T_{t}}\left(x\right)dx,\\ & = & -\int_{0}^{t}\sum_{i=1}^{n}p_{i}\frac{\Gamma (n+1)}{\Gamma (i)\Gamma (n-i+1)}{\left(\frac{F(t-x)}{F(t)}\right)}^{i-1}{\left(1-\frac{F(t-x)}{F(t)}\right)}^{n-i}\frac{f(t-x)}{F(t)}\\ & \times & \log\left(\sum_{i=1}^{n}p_{i}\frac{\Gamma (n+1)}{\Gamma (i)\Gamma (n-i+1)}{\left(\frac{F(t-x)}{F(t)}\right)}^{i-1}{\left(1-\frac{F(t-x)}{F(t)}\right)}^{n-i}\frac{f(t-x)}{F(t)}\right)dx\\ & = & -\int_{0}^{t}\sum_{i=1}^{n}p_{i}\frac{\Gamma (n+1)}{\Gamma (i)\Gamma (n-i+1)}{\left(F_{t}\left(z\right)\right)}^{i-1}{\left(1-F_{t}\left(z\right)\right)}^{n-i}f_{t}\left(z\right)\\ & \times & \log\left(\sum_{i=1}^{n}p_{i}\frac{\Gamma (n+1)}{\Gamma (i)\Gamma (n-i+1)}{\left(F_{t}\left(z\right)\right)}^{i-1}{\left(1-F_{t}\left(z\right)\right)}^{n-i}f_{t}\left(z\right)\right)dx\\ & = & H\left(V\right)-\sum_{i=1}^{n}p_{i}\int_{0}^{1}g_{i}\left(u\right)\log f_{t}\left(F_{t}^{-1}\left(u\right)\right)du. \end{array}$$

The last equality is obtained by changing the variable of $u=F_{t}\left(z\right)$, and the proof is then completed. □

It is important to keep in mind that Equation (8) expresses the entropy of $T_{t}$ as the sum of two terms, where the first term does not depend on the distribution of past life-times, while the second term depends on the distribution of the past life-times of the component. If $T_{t}=\left[t-T|X_{n:n}\leq t\right]$ stands for the past life-time of the coherent system under the condition that at time t, all components of the system have failed, then $\bar{H}\left(T_{t}\right)$ calculates the expected amount of uncertainty induced by the conditional density of t−T, as long as $X_{n:n}\leq t,$ on the predictability of the past life-time of the system. Especially if we consider an *i*-out-of-*n* system with the system signature $p=(0,\ldots ,0,1_{i},0,\ldots ,0),\;i=1,2,\cdots ,n$, then Equation (9) to

(11) $$\bar{H}\left(T_{t}^{i}\right)=H\left(U_{i:n}\right)-E\left[\log f_{t}\left(F_{t}^{-1}\left(U_{i:n}\right)\right)\right],$$

for all t>0. The next theorem is a direct consequence of Theorem 1 that uses the property that the reversed hazard rate of *X* is decreasing. Recall that the random life-time *X* belongs to the class of the decreasing reversed hazard rate (DRHR) if τ(x) is a decreasing function of x>0.

**Theorem 2.**  *If X is DRHR, then $\bar{H}\left(T_{t}\right)$ is increasing in t.*

**Proof.**  Through the identity $f_{t}\left(F_{t}^{-1}\left(x\right)\right)=x\tau_{t}\left(F_{t}^{-1}\left(x\right)\right),$ Equation (9) can be rewritten as

(12) $$\begin{array}{c} \bar{H}\left(T_{t}\right)=H\left(V\right)-\sum_{i=1}^{n}p_{i}\left[\psi \left(n-i+1\right)-\psi \left(n+1\right)\right]-\sum_{i=1}^{n}p_{i}E\left[\log\tau_{t}\left(F_{t}^{-1}\left(U_{i:n}\right)\right)\right]. \end{array}$$

It is plain to verify that $F_{t}^{-1}\left(u\right)=F^{-1}\left(uF\left(t\right)\right),$ for all 0<u<1, and hence,

$$\begin{array}{ccc} \tau_{t}\left(F_{t}^{-1}\left(u\right)\right) & = & \tau (F^{-1}\left(uF\left(t\right)\right)),\;0<u<1. \end{array}$$

If $t_{1}\leq t_{2}$, then $F^{-1}\left(uF\left(t_{1}\right)\right)\leq F^{-1}\left(uF\left(t_{2}\right)\right)$. Consequently, when F is DRHR, then

$$E\left[\log\tau \left(F^{-1}\left(U_{i:n}F\left(t_{1}\right)\right)\right)\right]\geq E\left[\log\tau \left(F^{-1}\left(U_{i:n}F\left(t_{2}\right)\right)\right)\right].$$

Using (12), the proof is then completed. □

The next example deals with a situation where Theorems 1 and 2 are applied.

**Example 1.**  Consider a coherent system with the signature p=(0,2/3,1/3). It follows that H(V)=−0.05757. Given the distributions of the components’ life-times, the Relation (9) can be used to determine the precise value of $H(T_{t})$. Let us assume the following life-time distributions for this purpose.

**Figure 1:**
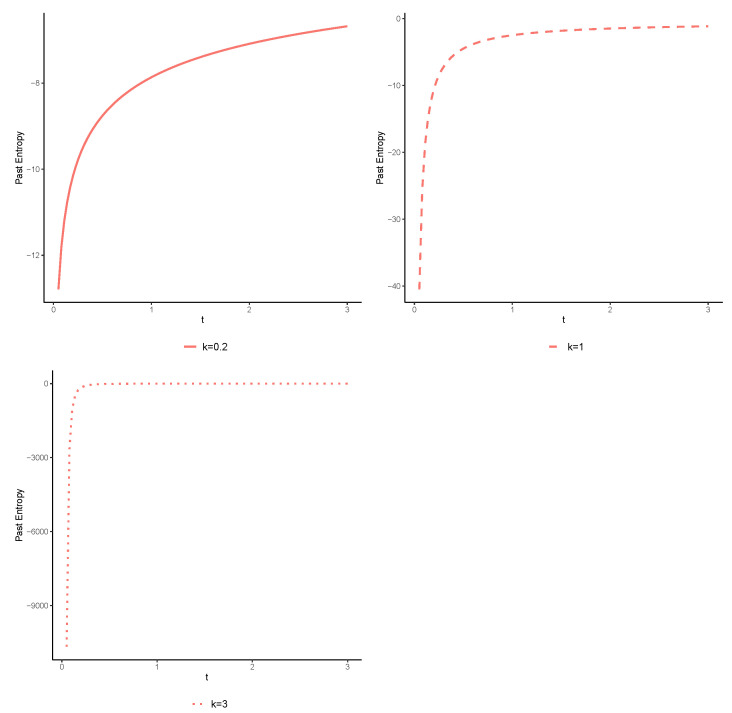
The exact value of $\bar{H}\left(T_{t}\right)$ for various values of *k*, as demonstrated in Part (b) of Example 1.

(a) Let *X* be uniformly distributed in [0,1]. It holds that
  $$E\left[\log f_{t}\left(F_{t}^{-1}\left(U_{i:n}\right)\right)\right]=-\log\left(t\right),$$
  for all i=1,2,3,4. From (8), we immediately obtain
$$\bar{H}\left(T_{t}\right)=-0.05757+\log\left(t\right).$$
  It is seen that the entropy of $T_{t}$ is an increasing function of time t. We note that the uniform distribution has the DRHR property, and therefore, $\bar{H}\left(T_{t}\right)$ is an increasing function of time *t*, as we expected based on Theorem 1.
(b) Let us assume that *X* follows the cdf
  $$F\left(x\right)=e^{-x^{-k}},\;x>0,\;k>0.$$
  One can see that
  $$E\left[\log f_{t}\left(F_{t}^{-1}\left(U_{i:n}\right)\right)\right]=\log\left(k\right)+E\left[\log U_{i:n}\right]+\frac{k+1}{k}E\left[\log\left(t^{-k}-\log\left(U_{i:n}\right)\right)\right],$$
  for all i=1,2,3,4. Upon recalling (9), we obtain
$$\bar{H}\left(T_{t}\right)=1.0257-\log\left(k\right)-\frac{k+1}{k}\sum_{i=1}^{n}p_{i}E\left[\log\left(t^{-k}-\log\left(U_{i:n}\right)\right)\right],$$
  for all t>0. For several choices of *k*, we have shown the exact value of $\bar{H}\left(T_{t}\right)$ with respect to time *t* in Figure 1. It is obvious that $\bar{H}\left(T_{t}\right)$ is an increasing function of time *t* for all k>0 since *X* is DRHR, as can follow from Theorem 1.

The duality of a system is a useful concept for technical reliability, which makes it possible to reduce the computational complexity for determining the signatures of all coherent systems of a given size by about half. Kochar et al. [15] have proposed a duality relation that exists between the signature of a system and that of its dual. If $p=(p_{1},\cdots ,p_{n})$ denotes the signature a coherent system with life-time *T* has, then the signature of its dual system with life-time $T^{D}$ is given by $p^{D}=\left(p_{n},\cdots ,p_{1}\right)$. In the following theorem, we apply the duality property to simplify the calculation of the past entropy for coherent systems. First, we need the following the lemma that is well-known as the Müntz–Szász theorem, and one can find it in [16].

**Lemma 1.**  *If ϕ(x) is a continuous function of [0,1], such that $\int_{0}^{1}x^{n}\phi \left(x\right)dx=0$ for all n≥0, then ϕ(x)=0 for any x∈[0,1].*

**Theorem 3.**  *Let $T_{t}$ be the life-time of a coherent system with signature p consisting of n i.i.d. components. Then, $H\left(T_{t}\right)=H\left(T_{t}^{D}\right)$ for all p and all n, if and only if $f_{t}\left(F_{t}^{-1}\left(u\right)\right)=f_{t}\left(F_{t}^{-1}\left(1-u\right)\right)$ satisfies for all 0<u<1 and t.*

**Proof.**  It is worth noting that Theorem 2.2 of Toomaj and Doostparast [17] asserts the equality of entropies between *V* and $V^{D}$, i.e., $H\left(V\right)=H\left(V^{D}\right)$. To prove sufficiency, let us assume that $f_{t}\left(F_{t}^{-1}\left(u\right)\right)=f_{t}\left(F_{t}^{-1}\left(1-u\right)\right)$ for all 0<u<1. It is worth noting that $g_{i}\left(1-u\right)=g_{n-i+1}\left(u\right)$ for all i=1,…,n and 0<u<1. Consequently, utilizing (9), we obtain that:

$$\begin{array}{ccc} -\int_{0}^{1}g_{V^{D}}\left(u\right)\log f_{t}\left(F_{t}^{-1}\left(u\right)\right)du & = & -\int_{0}^{1}\left(\sum_{i=1}^{n}p_{n-i+1}g_{i}\left(u\right)\right)f_{t}\left(F_{t}^{-1}\left(u\right)\right)du\\ & = & -\int_{0}^{1}\left(\sum_{r=1}^{n}p_{r}g_{n-r+1}\left(u\right)\right)\log f_{t}\left(F_{t}^{-1}\left(u\right)\right)du\\ & = & -\int_{0}^{1}\left(\sum_{r=1}^{n}p_{r}g_{r}\left(1-u\right)\right)\log f_{t}\left(F_{t}^{-1}\left(1-u\right)\right)du\\ & = & -\int_{0}^{1}\left(\sum_{r=1}^{n}p_{r}g_{r}\left(u\right)\right)\log f_{t}\left(F_{t}^{-1}\left(u\right)\right)du\\ & = & -\int_{0}^{1}g_{V}\left(u\right)\log f_{t}\left(F_{t}^{-1}\left(u\right)\right)du, \end{array}$$

and this completes the proof by recalling Equation (8). For necessity, $H\left(T_{t}\right)=H\left({T_{t}}^{D}\right)$ holds for all p and all n. Let p=(1,0,…,0). So, it follows from (9) that the assumption $H\left(T_{t}\right)=H\left({T_{t}}^{D}\right)$ is equivalent to

$$\begin{array}{ccc} -\int_{0}^{1}g_{n}\left(u\right)\log f_{t}\left(F_{t}^{-1}\left(u\right)\right)du & = & -\int_{0}^{1}g_{1}\left(u\right)\log f_{t}\left(F_{t}^{-1}\left(u\right)\right)du\\ & = & -\int_{0}^{1}g_{n}\left(1-u\right)\log f_{t}\left(F_{t}^{-1}\left(u\right)\right)du, \end{array}$$

where the last equality is obtained by noting that $g_{1}\left(u\right)=g_{n}\left(1-u\right),\;0<u<1.$ Putting v=1−u in the right side of the above equation leads to

$$\begin{array}{ccc} -\int_{0}^{1}g_{n}\left(u\right)\log f_{t}\left(F_{t}^{-1}\left(u\right)\right)du & = & -\int_{0}^{1}g_{n}\left(u\right)\log f_{t}\left(F_{t}^{-1}\left(1-u\right)\right)du. \end{array}$$

Thus, we obtain

$$\begin{array}{ccc} \int_{0}^{1}g_{n}\left(u\right)\left[\log f_{t}\left(F_{t}^{-1}\left(u\right)\right)-\log f_{t}\left(F_{t}^{-1}\left(1-u\right)\right)\right]du & = & \int_{0}^{1}{\left(1-u\right)}^{n-1}\log\frac{f_{t}\left(F_{t}^{-1}\left(u\right)\right)}{f_{t}\left(F_{t}^{-1}\left(1-u\right)\right)}du\\ & = & \int_{0}^{1}u^{n-1}\log\frac{f_{t}\left(F_{t}^{-1}\left(1-u\right)\right)}{f_{t}\left(F_{t}^{-1}\left(u\right)\right)}du=0. \end{array}$$

Hence $f_{t}\left(F_{t}^{-1}\left(1-u\right)\right)=f_{t}\left(F_{t}^{-1}\left(u\right)\right)$ due to Lemma 1, and this concludes the proof. □

An immediate consequence of the above theorem is given for the *i*-out-of-*n* systems.

**Corollary 1.**  *Let $T_{t}^{i}$ be the life-time of an i-out-of-n system consisting of n i.i.d. components. Then, $\bar{H}\left(T_{t}^{i}\right)=\bar{H}\left(T_{t}^{n-i+1}\right)$ for all n and i=1,2,…,n/2 if n is even and i=1,2,…,(n−1)/2 if n is odd, if and only if $f_{t}\left(F_{t}^{-1}\left(u\right)\right)=f_{t}\left(F_{t}^{-1}\left(1-u\right)\right)$ satisfies for all 0<u<1 and t.*

## 3. Bounds for the Past Entropy

Hereafter, we provide several useful bounds for $\bar{H}\left(T_{t}\right)$ by using the concept of the system signature. For the first bound, we use the notion of Kullback–Leibler (KL) discrimination information. We recall that the KL discrimination information between two random variables *X* and *Y* with pdfs *f* and g, respectively, is given by

(13) $$K\left(X:Y\right)=\int_{0}^{\infty }f\left(x\right)\log\frac{f(x)}{g(x)}dx=-H\left(X\right)+H\left(X,Y\right),$$

where H(X,Y)=−E(logg(X)) is known as the inaccuracy between *f* and g.

**Theorem 4.**  *Let $T_{t}$ denote the past life-time of a coherent system consisting of n i.i.d. components’ life-times $X_{1},\cdots ,X_{n}$ having the common pdf f in which, at time t, all components of the system have failed. Then, we have*

(14) $$\begin{array}{c} {\bar{H}}_{L}\left(T_{t}\right)\leq \bar{H}\left(T_{t}\right)\leq {\bar{H}}_{U}\left(T_{t}\right), \end{array}$$

where ${\bar{H}}_{L}\left(T_{t}\right)=\sum_{i=1}^{n}p_{i}\bar{H}\left(T_{t}^{i}\right)$ and ${\bar{H}}_{U}\left(T_{t}\right)={\bar{H}}_{L}\left(T_{t}\right)+\sum_{i=1}^{n}p_{i}K\left(U_{i:n}:U_{j^{*}:n}\right)$ for all t>0.

**Proof.**
  For the lower bound, since the differential entropy is a concave function of the density function, we can find a lower bound for the entropy of $T_{t}$ given by the following representation:

(15) $$\begin{array}{c} \bar{H}\left(T_{t}\right)\geq H_{L}\left(T_{t}\right)=\sum_{i=1}^{n}p_{i}\bar{H}\left(T_{t}^{i}\right). \end{array}$$

Moreover, the upper bound can be obtained by noting that the Kullback–Leibler (KL) discrimination information is a non-negative measure. Thus, we have

$$K\left(T_{t}:T_{t}^{j}\right)=-\bar{H}\left(T_{t}\right)+\bar{H}\left(T_{t},T_{t}^{j}\right)\geq 0.$$

So, one can obtain

(16) $$\bar{H}\left(T_{t}\right)\leq {\bar{H}}_{U}\left(T_{t}\right)=\min_{1\leq j\leq n}\bar{H}\left(T_{t},T_{t}^{j}\right).$$

The upper bound (16) can be rewritten as

(17) $$\begin{array}{ccc} \bar{H}\left(T_{t},T_{t}^{j}\right) & = & \sum_{i=1}^{n}p_{i}\bar{H}\left(T_{t}^{i},T_{t}^{j}\right)\\ & = & H_{L}\left(T_{t}\right)+\sum_{i=1}^{n}p_{i}K\left(U_{i:n}:U_{j:n}\right), \end{array}$$

where

$$K\left(U_{i:n}:U_{j:n}\right)=\log\left(\frac{\Gamma (j)\Gamma (n-j+1)}{\Gamma (i)\Gamma (n-i+1)}\right)+\left(i-j\right)\left[\psi (i)-\psi (n-i+1)\right],$$

denotes the Kullback–Leibler divergence of beta distributions (see [18] for details). The second equality in (17) is obtained by noting that the KL function is invariant under one-to-one transformations. If we assume that $j^{*}=\underset{1\leq j\leq n}{\arg\min}\sum_{i=1}^{n}p_{i}K\left(U_{i:n}:U_{j:n}\right)$, then

(18) $${\bar{H}}_{U}\left(T_{t}\right)={\bar{H}}_{L}\left(T_{t}\right)+\sum_{i=1}^{n}p_{i}K\left(U_{i:n}:U_{j^{★}:n}\right),$$

and the proof is then completed. □

We remark that by recalling Equation (11), the lower bound can be rewritten as

(19) $${\bar{H}}_{L}\left(T_{t}\right)=\sum_{i=1}^{n}p_{i}H\left(U_{i:n}\right)-\sum_{i=1}^{n}p_{i}E\left[\log f_{t}\left(F_{t}^{-1}\left(U_{i:n}\right)\right)\right].$$

It is worth pointing out that using Equation (11), expression (9) can be rewritten as

(20) $$\begin{array}{ccc} \bar{H}\left(T_{t}\right) & = & H\left(V\right)-\sum_{i=1}^{n}p_{i}H\left(U_{i:n}\right)+\sum_{i=1}^{n}p_{i}\bar{H}\left(T_{t}^{i}\right)\\ & = & H\left(V\right)-H_{L}\left(V\right)+{\bar{H}}_{L}\left(T_{t}\right), \end{array}$$

where $H_{L}\left(V\right)=\sum_{i=1}^{n}p_{i}H\left(U_{i:n}\right).$ It is worth noting that the difference between the past entropy and the lower bound of $T_{t},$ i.e., $\bar{H}\left(T_{t}\right)-H_{L}\left(T_{t}\right)$ is distribution free and depends only on the system signature. For further information about the bounds and to obtain the optimal index $j^{*},$ we refer the reader to [10,19].

Numerous authors have investigated the characteristics of coherent systems with various distribution components including Murthy and Jiang [20], Jiang et al. [21], Castet and Saleh [22], and Qiu et al. [23], as well as the references therein. To compare the bounds derived in Theorems 4 and 5, we present an example of a coherent system with power distribution components.

**Example 2.**  Consider a coherent system having signature p=(0,2/3,1/3). It is easy to see that H(V)=−0.0874. Moreover, we can obtain $j^{★}=2$ (see e.g., [24]). The exact value of $H(T_{t})$ can be computed using Relation (9) when the component life-time distributions are given. Let us denote the life-time of each component by X. We assume that *X* is a power distribution random variable, with the pdf given by

$$f\left(x\right)=kx^{k-1},\;0<x<1,$$

for all k>0. It is plain to observe that

$$-E\left[\log f_{t}\left(F_{t}^{-1}\left(U_{i:n}\right)\right)\right]=\log\left(\frac{t}{k}\right)-\frac{k-1}{k}\left[\psi (n-i+1)-\psi (n+1)\right],$$

for all i=1,2,3. From (8), we immediately obtain

(21) $$\bar{H}\left(T_{t}\right)=-0.0874-\left(\frac{k-1}{k}\right)1.1666+\log\left(\frac{t}{k}\right).$$

Alternatively, from Equation (19), the lower bound is given as:

(22) $${\bar{H}}_{L}\left(T_{t}\right)=-0.2273-\left(\frac{k-1}{k}\right)1.1666+\log\left(\frac{t}{k}\right).$$

The upper bound can be obtained by recalling Equation (18) as follows:

(23) $${\bar{H}}_{U}\left(T_{t}\right)=0.0416-\left(\frac{k-1}{k}\right)1.1666+\log\left(\frac{t}{k}\right).$$

**Figure 2:**
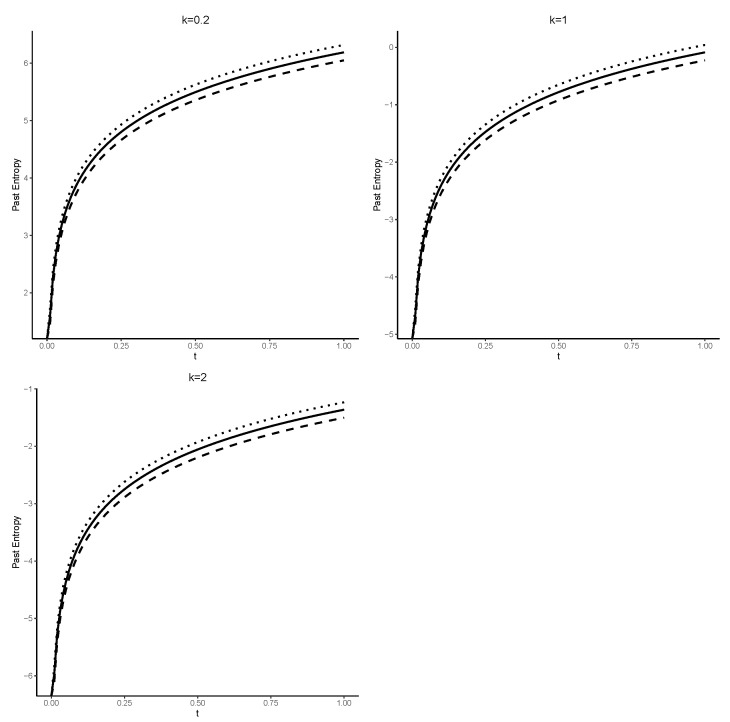
The exact value of $\bar{H}\left(T_{t}\right)$ given in Equation (21) (solid line) along with the lower bound given in Equation (22) (dashed line) and the upper bound given in Equation (23) (dotted line) for different values of *k*, as demonstrated in Example 2.

The entropy of $T_{t}$ is a monotonically increasing function of time *t*. We note that the power distribution possesses the DRHR property, thus, as expected due to Theorem 1, $\bar{H}\left(T_{t}\right)$ is also an increasing function of time *t*. Figure 2 displays the exact value of $\bar{H}\left(T_{t}\right)$ together with the lower and upper bounds computed as described above for various values of *k*. As predicted by Theorem 1, it is evident that $\bar{H}\left(T_{t}\right)$ monotonically increases with respect to time *t* for all k>0, since *X* is DRHR.

Another useful lower bound can be obtained in the next theorem.

**Theorem 5.**  *By assuming that the conditions in Theorem 4 hold, one obtains*

(24) $$\begin{array}{ccc} \bar{H}\left(T_{t}\right) & \leq & {\bar{H}}_{L}\left(T_{t}\right)-H_{L}\left(V\right), \end{array}$$

*for all t>0.*

**Proof.**  Due to Lemma 4.1 of Toomaj et al. [24], it holds that H(V)≤0. Upon recalling Equation (20), the proof is then completed. □

The following theorem compares the past entropies of two coherent systems that have distinct structures and the same component life-times.

**Theorem 6.**  *Let $T_{1,t}=\left[t-T_{1}|X_{n:n}\leq t\right]$ and $T_{2,t}=\left[t-T_{2}|X_{n:n}\leq t\right]$ represent the past life-times in two coherent systems with signatures $p_{1}$ and $p_{2}$, respectively, so that $p_{1}\leq_{st}p_{2}$. Let the system’s components be i.i.d. with the common cdf F. Then, for t>0,*

**(i)**  *if $H\left(V_{1}\right)\geq H\left(V_{2}\right)$ and $f_{t}\left(F_{t}^{-1}\left(u\right)\right)$ increases in u for all t>0, then $\bar{H}\left(T_{1,t}\right)\geq \bar{H}\left(T_{2,t}\right)$.*
**(ii)**  *if $H\left(V_{1}\right)\leq H\left(V_{2}\right)$ and $f_{t}\left(F_{t}^{-1}\left(u\right)\right)$ decreases in u for all t>0, then $\bar{H}\left(T_{1,t}\right)\leq \bar{H}\left(T_{2,t}\right)$.*

**Proof.**  (i) First, it should be noted that the following equation can be used to rewrite Equation (9):

(25) $$\bar{H}\left(T_{i,t}\right)-H\left(V_{i}\right)=-\int_{0}^{1}g_{V_{i}}\left(u\right)\log\left(f_{t}\left(F_{t}^{-1}\left(u\right)\right)\right)du,\;\left(i=1,2\right).$$

Assumption $p_{1}\leq_{st}s_{2}$ implies $V_{1}\leq_{st}V_{2}.$ So, we obtain

(26) $$\begin{array}{ccc} -\int_{0}^{1}g_{V_{1}}\left(u\right)\log\left(f_{t}\left(F_{t}^{-1}\left(u\right)\right)\right)du & \geq & -\int_{0}^{1}g_{V_{2}}\left(u\right)\log\left(f_{t}\left(F_{t}^{-1}\left(u\right)\right)\right)du, \end{array}$$

in which the inequality in (26) is derived in spirit of the implication that $V_{1}\leq_{st}V_{2}$ implies $E\left[\pi \left(V_{1}\right)\right]\geq E\left[\pi \left(V_{2}\right)\right]$ for all decreasing functions of π. Therefore, Relation (25) gives

$$\bar{H}\left(T_{1,t}\right)-H\left(V_{1}\right)\geq \bar{H}\left(T_{2,t}\right)-H\left(V_{2}\right),$$

or equivalently

$$\bar{H}\left(T_{1,t}\right)-\bar{H}\left(T_{2,t}\right)\geq H\left(V_{1}\right)-H\left(V_{2}\right)\geq 0,$$

where the last inequality is obtained from the assumption and hence the theorem. Part (ii) is analogously proven. □

The following example supplies a situation to apply to Theorem 6.

**Figure 3:**
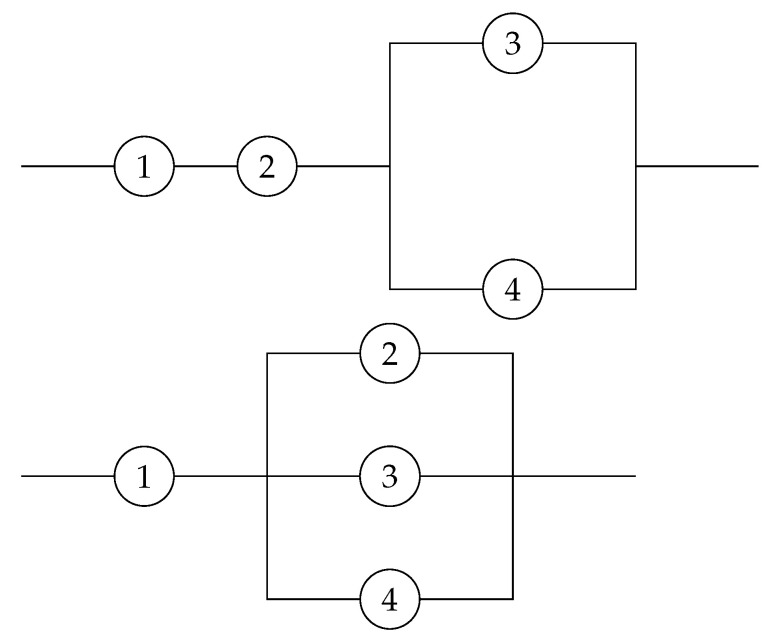
Two coherent systems that have signatures with likelihood ratio ordering properties.

**Example 3.**  We take into account two coherent systems with four components shown in Figure 3 with past life-times $T_{1,t}=\left[t-T_{1}|X_{4:4}\leq t\right]$ (left panel) and $T_{2,t}=\left[t-T_{2}|X_{4:4}\leq t\right]$ (right panel). It is easily identified that $p_{1}=\left(\frac{1}{2},\frac{1}{2},0,0\right)$ and $p_{2}=\left(\frac{1}{4},\frac{1}{4},\frac{1}{2},0\right),$ respectively. Further, we can plainly see that $H(V_{1})=-0.2970$ and that $H(V_{2})=-0.0575$, hence, $H\left(V_{1}\right)\leq H\left(V_{2}\right).$ Moreover, we have $p_{1}\leq_{st}p_{2}.$ Suppose that the component life-times are i.i.d. with the standard exponential distribution with the cdf $F\left(t\right)=1-e^{-t},\;t>0.$ It is easily seen that

$$f_{t}\left(F_{t}^{-1}\left(u\right)\right)=\frac{1-u(1-e^{-t})}{1-e^{-t}},\;t>0,$$

for all 0<u<1. Obviously, $f_{t}\left(F_{t}^{-1}\left(u\right)\right)$ is a decreasing function of *u* for all t>0. Hence, due to Theorem 6, it holds that $\bar{H}\left(T_{1,t}\right)\leq \bar{H}\left(T_{2,t}\right)$ for all t>0.

In the next theorem, we use the concept of duality to reduce the calculation of the past entropy of coherent systems. We recall that $\leq_{st}$ stands for the stochastic order (see Shaked and Shanthikumar [25]).

**Corollary 2.**  *Let $T_{t}=\left[t-T|X_{n:n}\leq t\right]$ represent the past life-time of a coherent system with signature vectors p and let $T_{t}^{D}=\left[t-T^{D}|X_{n:n}\leq t\right]$ be its dual with signature $s^{D}$ consisting of i.i.d. component life-time with the common cdf F. Let also $p\leq_{st}p^{D}.$ Then,*

**(i)**  *if $f_{t}\left(F_{t}^{-1}\left(u\right)\right)$ increases in u for all t>0, then $\bar{H}\left(T_{t}\right)\geq \bar{H}\left(T_{t}^{D}\right)$.*
**(ii)**  *if $f_{t}\left(F_{t}^{-1}\left(u\right)\right)$ decreases in u for all t>0, then $\bar{H}\left(T_{t}\right)\geq \bar{H}\left(T_{t}^{D}\right)$.*

## 4. Jensen–Shannon Divergence of System

This section presents an analytical expression for the Jensen–Shannon (JS) divergence of the past life-time of a coherent system. Specifically, we demonstrate that the JS divergence of the proposed past life-time provides an information criterion for comparing systems based solely on their designs, independent of the parent distribution function *F* of the system. Drawing on earlier findings by Asadi et al. [18], the JS divergence of the mixture given by Equation (5) can be defined as follows:

(27) $$\begin{array}{ccc} JS(T_{t}:T_{t}^{1},\ldots ,T_{t}^{n};p) & = & JS\left(p\right)=\bar{H}\left(T_{t}\right)-\sum_{i=1}^{n}p_{i}\bar{H}\left(T_{t}^{i}\right)\\ & = & H\left(V\right)-\sum_{i=1}^{n}p_{i}H\left(U_{i:n}\right). \end{array}$$

By recalling Equation (11), we easily obtain

$$\sum_{i=1}^{n}p_{i}\bar{H}\left(T_{t}^{i}\right)=\sum_{i=1}^{n}p_{i}H\left(U_{i:n}\right)-\sum_{i=1}^{n}p_{i}E\left[\log f_{t}\left(F_{t}^{-1}\left(U_{i:n}\right)\right)\right].$$

Upon recalling the above relation and (9), the third equality in (27) is easily obtained. It is worth pointing out that (27) does not depend on time *t* and the common cdf *F*, and it solely depends on the design of the system signature that coincides with the results given by [18].Therefore, all given results in that paper are also held.

It is evident from (27) that the past life-time entropy concerning the coherent system can be written in terms of JS divergence as follows:

(28) $$\bar{H}\left(T_{t}\right)=JS\left(p\right)+\sum_{i=1}^{n}p_{i}\bar{H}\left({T_{t}}^{i}\right),$$

for all t>0. This representation is useful and interesting since it relates the entropy of $T_{t}$ to the JS divergence as well as a weighted sum of the past entropy of order statistics. From the results of Toomaj et al. [10], we have the following useful results for which its proof is omitted.

**Theorem 7.**  *For a given coherent system with signature s and dual system with signature $s^{D},$ we have*

$$JS\left(p\right)=JS\left(p^{D}\right).$$

Boundaries play a crucial role in many areas of research; therefore, much attention has been paid to the study of the acquisition of boundaries presented in the literature. Asadi et al. [18] proposed an approach to computing upper bounds for the Jensen–Shannon (JS) divergence. Specifically, let N be a random variable with a probability mass function $p=(p_{1},\ldots ,p_{n})$, where $p_{i}=P\left(N=n_{i}\right)$ and $n_{i}=1,2,\ldots ,n,$ represent the number of failures of components fatal to the system. The Shannon entropy of the signature vector $H\left(N\right)=H\left(p\right)=-\sum_{i=1}^{n}p_{i}\log p_{i}$ measures the uncertainty associated with the failure of the system due to the failure of its components. Asadi et al. [18] have derived a primitive upper bound for the JS divergence related to the Shannon entropy of the signature vector given by:

0≤JS(p)≤H(p).

The above representation allows for us to obtain bounds for $\bar{H}\left(T_{t}\right)$ in terms of the entropy of the signature vector as follows:

(29) $$H_{L}\left(T_{t}\right)\leq \bar{H}\left(T_{t}\right)\leq H\left(p\right)+H_{L}\left(T_{t}\right),$$

where $H_{L}\left(T_{t}\right)=\sum_{i=1}^{n}p_{i}\bar{H}\left({T_{t}}^{i}\right).$ In an attempt to obtain an improved upper bound, Asadi et al. [18] obtained the following representation that is applicable for the general case of the JS divergence of mixture distributions. Namely, it holds that

(30) $$0\leq JS\left(p\right)\leq \sum_{i=1}^{n}\sum_{j=1}^{n}p_{i}p_{j}K\left(U_{i:n}:U_{j:n}\right).$$

Therefore, the second upper bound for $\bar{H}\left(T_{t}\right)$ can be obtained by substituting (30) in place of H(p) in (29).

## 5. Concluding Remarks

In recent years and decades, researchers in the field of information theory have become increasingly interested in developing measures that can be used to evaluate the degree of uncertainty in random variables. The phenomenon of uncertainty associated with the life-time of engineering systems is related to other aspects of the systems. For example, imagine a situation in which an inspection at time *t* by an operator makes it clear that a number of components that were functioning in a system have become inactive. The problem here is that an event has occurred in the past, but there is still uncertainty about the exact time at which the system or the components within it failed. The ability to assess predictability over the life-time of a system can be a valuable criterion in this regard. Differential Shannon entropy has proven to be an attractive measure for quantifying uncertainty in such situations. Assuming that each system component has failed at time *t*, we have established in this work an equation for the entropy of the life-time of a system. We have also investigated various properties of this proposed measure, including the determination of boundaries and partial orders between the past life-times of two coherent systems based on their entropy uncertainties using the concept of system signature. To demonstrate the effectiveness of our approach, we give several examples of its application. Our results highlight the potential of this measure for assessing the predictability of system life-times and its usefulness for engineering applications.

## Data Availability

No new data were created or analyzed in this study. Data sharing is not applicable to this article.
